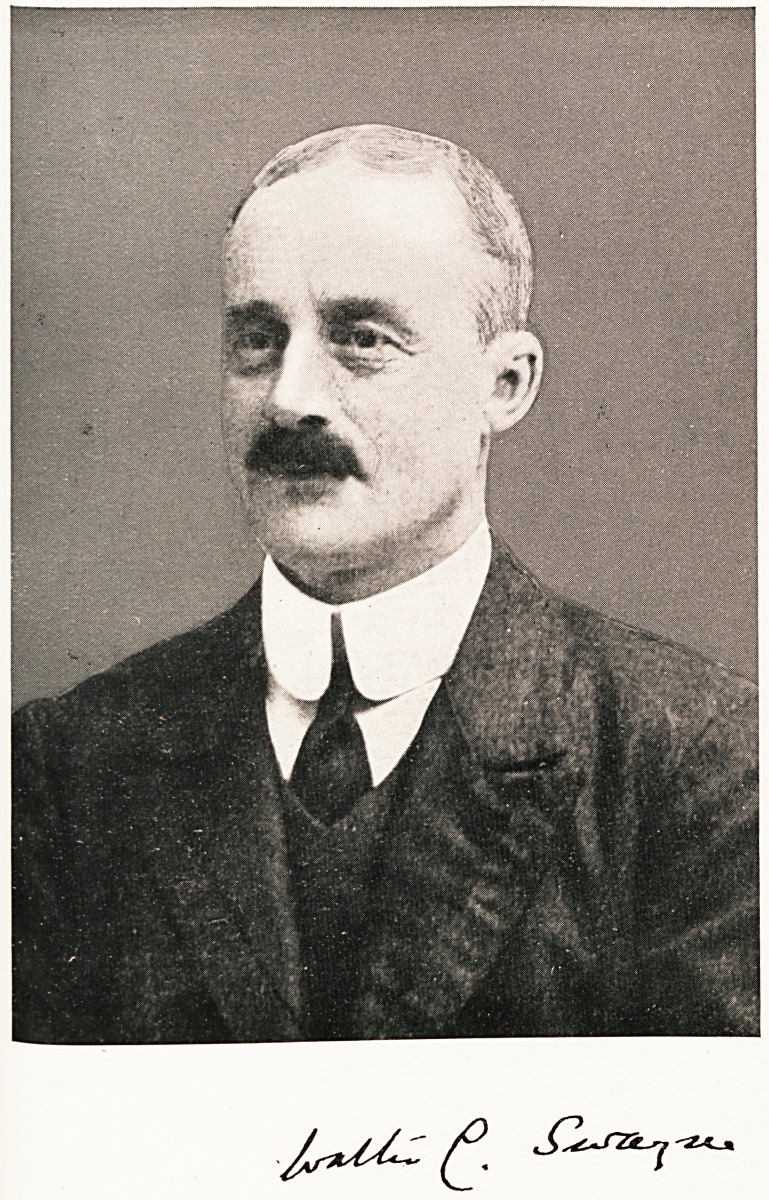# Walter Carless Swayne

**Published:** 1925

**Authors:** 


### Obituary.

### WALTER CARLESS SWAYNE, M.D., B.S. Lond., M.D., Ch.B. Bristol.

THE tragedy of Professor W. C. Swayne's untimely end lies not only in the particularly painful circumstances which resulted in his death, but in the great loss to the profession which he adorned by his unselfish devotion to the great work of founding an obstetric and gynæcological clinic worthy of a University school of medicine, and which his persistent and tactful determination of purpose most meritoriously attained and bequeathed to us.

Having just completed his work of examining at the University, he joined his son-in-law, Mr. R. D. Wreford Brown, and Mrs. Wreford Brown, his daughter, and his son, who was already with them, at their residence at Sellacks, near Hereford. Mr. Wreford Brown had been badly gassed at Cambrai, and further, as a legacy of his war service, suffered from a chronic recurring purulent otitis, and his mental condition was causing anxiety. The family party had seemed happy enough and all retired to bed, but about one o'clock in the morning Mr. Wreford Brown apparently became acutely deranged, fired revolver shots out of his bedroom window, "to attract attention," he said; then, going across the passage, he aroused Dr. Walter

Walter C. Swayne

Swayne, declaring the house was gassed, and in the darkness shot his father-in-law in the abdomen and in the hips. Young Swayne ran in and bravely secured his brother-in-law.

It was so characteristic of Walter Swayne that his first thought as he lay mortally wounded was to inquire if anyone else was hit. This somewhat isolated and remote district presented such difficulty in obtaining adequate assistance that Dr. Walter Swayne had expired several hours before his surgical colleague, who had hurried from Bristol as soon as the message got through, had arrived. Those of us who knew Mr. Wreford Brown's gentle and kindly disposition can join with his wife and their relations in attributing this terrible disaster to delusions apparently determined by conditions resulting from his war service.

W. C. Swayne, the eldest son of the late Mr. R. D. Swayne, of Tillington Court, Hereford, who had fourteen children, was born at Mathon, near Malvern, in 1862. He was educated at King Edward's School, Birmingham, and later came to live with his uncle, the late Joseph Griffiths Swayne, to enter the Bristol University College and General Hospital. In 1886 he went up to Guy's Hospital for the completion of his clinical work. After taking the double qualification of the Colleges, and in 1888 after graduating M.B. at the University of London with honours in forensic medicine, he was appointed Resident Obstetric Physician to Dr. Galabin and Dr. P. Horrocks, and shortly afterwards commenced to practise in Clifton, where he soon made his mark as an obstetrician. In 1891 he was appointed Obstetric Physician to the Bristol Royal Infirmary, a comparatively new post which had been initiated in 1887 with six beds for in-patients and an out-patient department. Dr. Swayne experienced at the Royal Infirmary the usual opposition to the development of the Gynæcological Department on modern lines. It was only some years later that the rules were altered to permit the Obstetric Physician performing abdominal operations, and Swayne's operating theatre was organised by him on the strictest antiseptic lines. His theatre "drill" was always irreproachable, and his skill as a diagnostician and operator led to a great expansion of the clinic.

It is worthy of note that although Swayne found himself opposing and opposed by his colleagues in his long struggle for the right of a gynæcologist to perform major operations his friendships with them never suffered. He was always " Dicky " Swayne to his opponents in the midst of hot controversy. In the end he succeeded in establishing a model department of midwifery both intern and extern and of gynæcology.

Dr. Swayne contributed many articles to medical journals on Obstetrics and Gynæcology. Some of his more important dealt with eclampsia and its bio-chemical problems. He often expressed his regret that he had begun to grow old in his profession before bio-chemistry was developed into a science, so that he lacked the requisite training for research in a subject that interested him so greatly.

Swayne was elected Professor of Midwifery in University College, Bristol, in 1902, and was continued in this Chair when the University was incorporated in 1909. He was an admirable teacher and a most loyal graduate of the new University, for he took the *ad eundem* degrees of M.D., Ch.B. at the earliest opportunity in Bristol on the strength of the degrees he already held from London University. He was an enthusiast in fostering a corporate spirit amongst the new graduates of Bristol, and was an ardent champion of the rights of Convocation, the representative body of the graduates. In the earliest days of the University he was unsparing in his criticism of the supreme authorities for their singular action in electing as professors all who had occupied Chairs in University College with the one exception of the Professor who had been Secretary of the Committee for Promoting the Foundation of the University. Swayne was keenly interested in the medical education of women, and the first woman resident at the Royal Infirmary was appointed to his Obstetric Department.

He was President of the Medico-Chirurgical Society in the year 1912–13, and his address then, like many of his original contributions to medical literature, was sound and erudite.

One of the most striking features in Swayne's professional career and in his character was his total lack of anything like jealousy for his younger colleagues and possible rivals in his own specialism, and it was with great pride that he used to watch the progress of his pupils Myles Phillips and King in Sheffield, Green-Armytage in India and Statham in Bristol.

It was largely due to his initiative that the M.Ch. is granted in Obstetrics, and nothing proves more strikingly his originality than the plan of making an operation by candidates for the mastership a part of the rational test of fitness for this the highest degree conferred in the speciality. Without being a prolific writer, he made many valuable contributions to the Bristol Medico-Chirurgical Society and other scientific bodies.

Outside of his profession Swayne's chief hobby lay in soldiering. He was a keen student of military history, and early in life joined the Gloucestershire Artillery Volunteers as a boy-trumpeter, and in after years was usually referred to as "Bombardier Dick." He eventually rose to the rank of Major in the Territorial Artillery and received the Volunteer Decoration after twenty years' service. At the commencement of the war he was transferred for a time to command the Bristol University O.T.C., and was instrumental in developing in connection with the O.T.C. an enthusiastic company of "volunteers" amongst men who were over military age. Subsequently he raised the third line of the South Midland R.F.A., and finally commanded a battery of New Zealand artillery on Salisbury Plain, which he had the satisfaction of conducting overseas to France, although, to his lasting regret, his age debarred him from remaining to serve with the battery at the front. In addition, Swayne was an ardent Freemason, and had been Master of St. Vincent Lodge in Bristol and a Grand Warden in the Province of Bristol. His chief sport in his later years was trout-fishing, and in his country wanderings with his rod he used to search out and study any remains of the Roman occupation of Britain that came in his way.

At the time of his death Dr. Swayne still occupied his Chair in the University and was in charge of the clinic for venereal diseases at the Bristol Royal Infirmary, a department which he had organised since the war with his customary thoroughness and administrative ability. He had retired from the active staff of the Infirmary as Obstetrician and Gynæcologist in

1924, and held the office of Honorary Consultant to that institution.

In his younger days he was a keen athlete, he played "Rugger" for Guy's and was in the Guy's Hospital rowing four. In later life he was recognised as an expert with the rod, well known on the Wylye and the Monnow.

Walter Carless Swayne was one of the most lovable and belovèd of Bristol's citizens, ever ready to help anyone who turned to him in trouble, and countless of his poorer patients and friends feel to-day the greatness of their loss.

In 1894 he married Louisa Margaret, daughter of the late Rev. R. F. Heath, and his widow and three daughters and a son, as well as his mother, survive him. To them we extend the deepest sympathy in their grievous loss, which his colleagues and many friends likewise feel so profoundly.

### BIBLIOGRAPHY OF THE LATE DR. W. C. SWAYNE.

"Abdominal Hysterectomy for Fibroids of the Uterus with Retroperitoneal Treatment of the Stump, and Notes of Two Cases," *Bris. Med.-Chir. J.*, 1900, xviii. 123–127.

"Cystic Fibroid of Uterus," *Ibid.*, p. 369.

"Specimen of Cystic Fibroid of the Uterus removed by Panhysterectomy," *Bris. Med.-Chir. J.*, 1903, xxi. 83.

"Sarcoma of the Body of the Uterus with Complete Inversion," *Tr. Obst. Soc. Lond.* (1902), 1903, xliv., 366–368.

"Case of Uterine Fibroid Removed by Panhysterectomy," *Tr. Obst. Soc. Lond.* (1903), 1904, xlv. 378–380.

"Case of Fibroid of the Vagina," *Tr. Obst., Soc. Lond.* (1903), 1904, xlv. 142.

"A Case of Cæserean Section for Obstruction of Labour by a Pelvic Tumour," *Brit. Med.-Chir. J.*, 1904, xxii. 120–123.

"Uterine Fibroids showing Sarcomatous Degeneration," *Proc. Roy. Soc. Med.*, 1907–8, i., Obst. and Gyn. Sect., 151–153.

"Abdominal Myomectomy during Pregnancy," *Ibid.*, 129–132.

"Chorionepithelioma," *Brit. M. J.*, 1907, ii. 440–443.

"Cerebral Lesions in Pregnancy and Parturition," *Bris. Med.-Chir. J.*, 1907, xxv. 209–213.

"The Problem of Medical Education," *Bris. Med.-Chir. J.*, 1912, xxx. 316–327.

"The Clinical Significance of Acidosis in Pregnancy," *Proc. Roy. Soc. Med.*, 1911–12, v., Obst. and Gyn. Sect., 315–324.

"Two Cases of Missed Labour," *Ibid.*, 165–168.

"The Clinical Significance of Acidosis in Pregnancy," *Med. Press and Circ.*, 1912, n.s. xciv. 58–61.

"Operations on the Uterus and Appendage during Pregnancy," *Bris. Med.-Chir. J.*, 1912, xxx. 31–36.

" Rectal Diverticula as a Causative Factor in Pelvic Inflammation. in Women," *Med. Press and Circ.*, 1917, civ. 313, *Bris. Med.-Chir. J.*, 1917, xxxv. 91–95.

" An Operation for the Cure of Prolapse and Cystocele," *Bris. Med.-Chir. J.*, 1919–20, xxxvii. 81–87.

" Quinine as an Abortifacient," *Lancet*, 1919, i. 841.

" Syphilis in Women and Children," *Brit. M. J.*, 1921, ii. 476–480.

" Rupture of the Uterus during first stage of Labour," *Surg. Gyn. and Obst.*, 1922, xxxiv. 257.

## Figures and Tables

**Figure f1:**